# Right ventricular dilatation: echocardiographic differential diagnosis

**DOI:** 10.1007/s10396-023-01399-4

**Published:** 2024-01-16

**Authors:** Michiyo Yamano, Tetsuhiro Yamano, Satoaki Matoba

**Affiliations:** Department of Cardiovascular Medicine, Graduate School of Medical Science, Kyoto Prefectural University of Medicine, Kajii-cho 465, Kawaramachi-Hirokoji, Kamigyo-ku, Kyoto, 602-8566 Japan

**Keywords:** Right ventricular dilatation, Right ventricular function, Transthoracic echocardiography

## Abstract

The initial means of detecting right ventricular (RV) dilatation is often transthoracic echocardiography (TTE), and once the presence of RV dilatation is suspected, there is the possibility of RV volume overload, RV pressure overload, RV myocardial disease, and even nonpathological RV dilatation. With respect to congenital heart disease with RV volume overload, defects or valvular abnormalities can be easily detected with TTE, with the exception of some diseases. Volumetric assessment using three-dimensional echocardiography may be useful in determining the intervention timing in these diseases. When the disease progresses in patients with pulmonary hypertension as a result of RV pressure overload, RV dilatation becomes more prominent than hypertrophy, and RV functional parameters predict the prognosis at this stage of maladaptive remodeling. The differential diagnosis of cardiomyopathy or comparison with nonpathological RV dilatation may be difficult in the setting of RV myocardial disease. The characteristics of RV functional parameters such as two-dimensional speckle tracking may help differentiate RV cardiomyopathy from other conditions. We review the diseases presenting with RV dilatation, their characteristics, and echocardiographic findings and parameters that are significant in assessing their status or intervention timing.

## Introduction

The right ventricle (RV) is 10–15% larger than the left ventricle (LV), as reported in a study using cardiac magnetic resonance (CMR) [1]. Transthoracic echocardiographic (TTE) assessment of RV dilatation has been widely reported and been often debated as to which parameter best correlates with the volume using CMR. We can calculate RV volumes with high accuracy using three-dimensional (3D) TTE, though it requires a specialized vendor and the technique is impractical for screening. Echocardiographic findings other than a dilated cavity may help in the differential diagnosis of RV dilatation due to the number of diseases that manifest with this condition. The aim of this review article is to summarize these diseases and to identify features that may be helpful in formulating a differential diagnosis.

### RV dilatation on TTE

The RV has a complex shape, forming a crescent around the LV, and therefore assumptions that apply to LV geometry do not apply to the RV. Although RV size measurements obtained with two-dimensional (2D) TTE and CMR were shown to be poorly correlated [2], 2D TTE is often the initial means of diagnosing RV dilatation. An RV-focused apical four-chamber view is recommended for measuring the linear diameter of the RV inflow, and a diameter > 41 mm at the basal level and > 35 mm at the midlevel indicates RV dilatation [3, 4], though we often diagnose RV dilatation based on the balance between the RV and LV [5]. 3D TTE allows for the assessment of RV volume without the need for geometric assumptions. Obtaining an adequate measurement of whole RV volume with the transducer is difficult due to factors such as inadequate assessment of the RV outflow tract and unsatisfactory near-field resolution [6, 7]; therefore, efforts are needed to obtain adequate images. Although the normal range of RV volumes yielded by 3D TTE varies depending on the published report, the guidelines published by the American Society of Echocardiography and the European Association of Cardiovascular Imaging state that the upper limits of the RV end-diastolic volume index are 87 mL/m^2^ in men and 74 mL/m^2^ in women, while those of the RV end-systolic volume index are 44 mL/m^2^ in men and 36 mL/m^2^ in women [3].

**Fig. 1:**
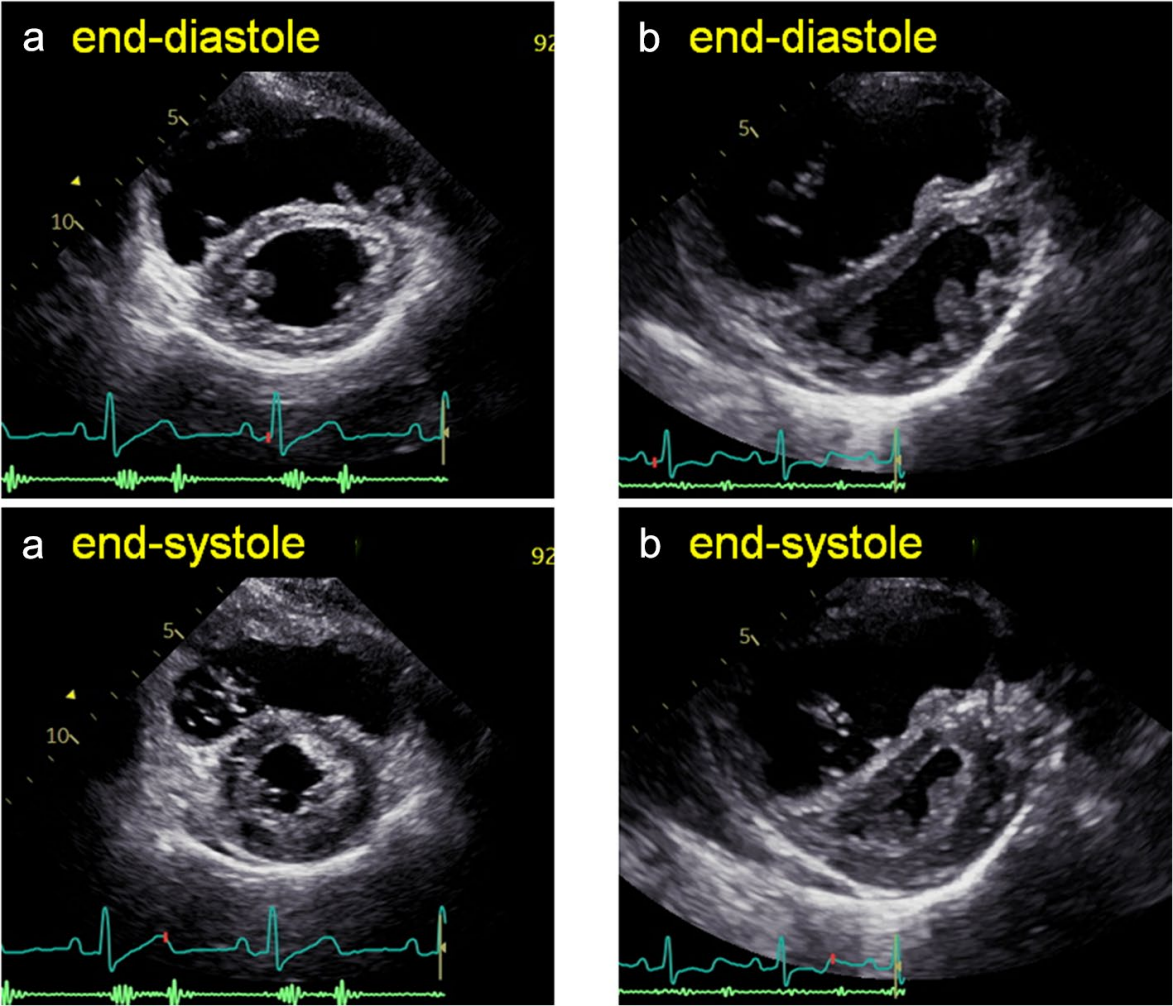
Septal flattening in patients with RV volume overload and RV pressure overload. **a** Septal flattening is observed only during diastole in a patient with RV volume overload. **b** Septal flattening is prominent during end-systole in a patient with RV pressure overload. *RV* right ventricular

Once RV dilatation is suspected on TTE, the causative factors include diseases associated with RV volume overload or pressure overload, as well as RV myocardial disease and nonpathological RV dilatation. Table 1 shows the causes of RV dilatation that we commonly encounter in the clinical setting and the commonly accepted or published findings of echocardiographic diagnosis and assessment. In distinguishing between RV volume overload and pressure overload, the findings on 2D TTE are helpful in most cases, along with clinical characteristics. The two conditions often demonstrate differences in interventricular septal morphology in the parasternal short-axis view, i.e., septal flattening is prominent during end-systole in patients with RV pressure overload, whereas it is observed during diastole in patients with volume overload (Fig. 1) [8]. A hypertrophied free wall and trabeculations may be observed in the RV with pressure overload [9], but prominent trabeculations are also observed in patients with RV volume overload.

**Table 1:** Causes of RV dilatation

**RV volume overload**	
Atrial level or preatrial shunt	
Secundum-type ASD	Defects can be visualized on TTE
Sinus venous-type ASD	Right sternal window or agitated saline contrast may be useful
Post ICR for TOF	
Pulmonary stenosis	Estimation of RV systolic pressure is important
Pulmonary regurgitation	The severity should be estimated based on multiple parameters Volumetric assessment with 3D TTE is useful
Tricuspid regurgitation	RV functional parameters are predictors after surgery [22, 23]
Pulmonary regurgitation	
**RV pressure overload**	
Pulmonary hypertension	In the stage of maladaptive remodeling, the RV enlarges and its contraction may be impaired [25]
RV outflow tract obstruction	
Pulmonary embolism	
**RV myocardial disease**	
ARVC	Parameters of 2D speckle tracking may detect it in the subclinical or electrical stage [33] Impaired contractility in basal RV segment [34]
Cardiac sarcoidosis	Basal LV longitudinal strain may be impaired [37]
RV myocardial infarction	Isolated RV infarction is a rare condition [43, 44]
**Nonpathological RV dilatation**	
Athlete’s heart	It is associated with LV remodeling [41]
Abnormality of the thoracic cage	RV dilatation is observed in some subjects with pectus excavatum [42]

*ARVC* arrhythmogenic right ventricular cardiomyopathy, *ASD* atrial septal defect, *ICR* intracardiac repair, *LV* left ventricular, *RV* right ventricular, *3D* three-dimensional, *TOF* tetralogy of Fallot, *TTE* transthoracic echocardiography, *2D* two-dimensional

## RV volume overload

### Atrial septal defect (ASD)

**Fig. 2:**
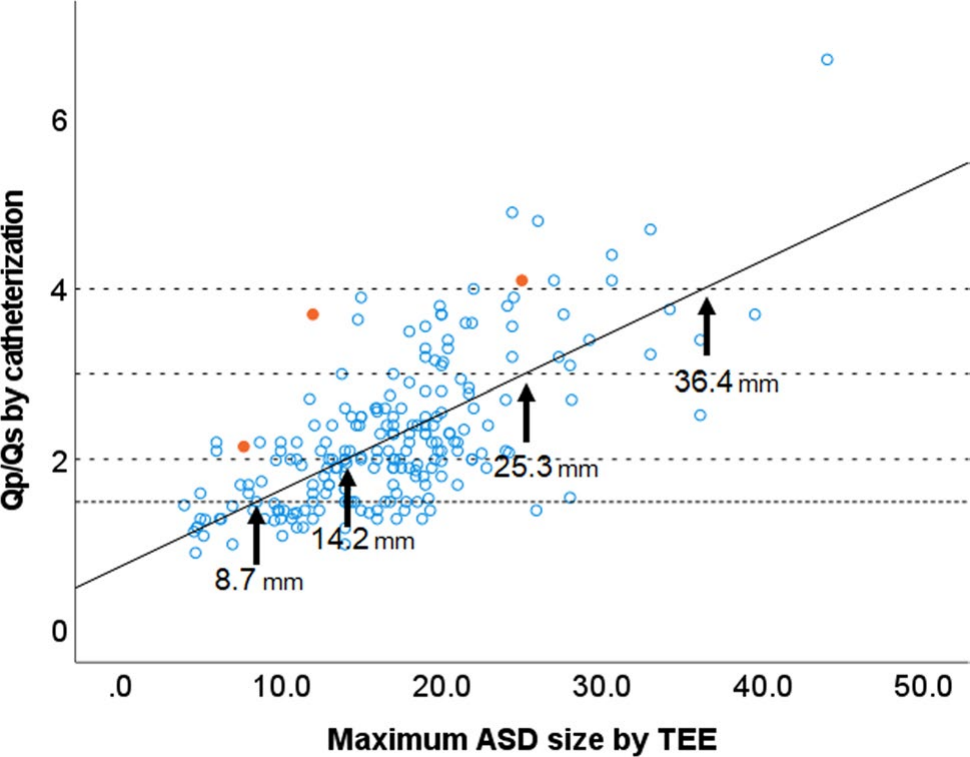
Correlation between maximum ASD size and pulmonary to systemic blood flow ratio. A significant relationship is observed between maximum ASD size and pulmonary to systemic blood flow ratio (Qp/Qs) (R = 0.68, *P* < 0.001). Orange dots represent patients with partial anomalous pulmonary venous return. ASD, atrial septal defect; TEE, transesophageal echocardiography; Qp/Qs, pulmonary to systemic blood flow ratio

**Fig. 3:**
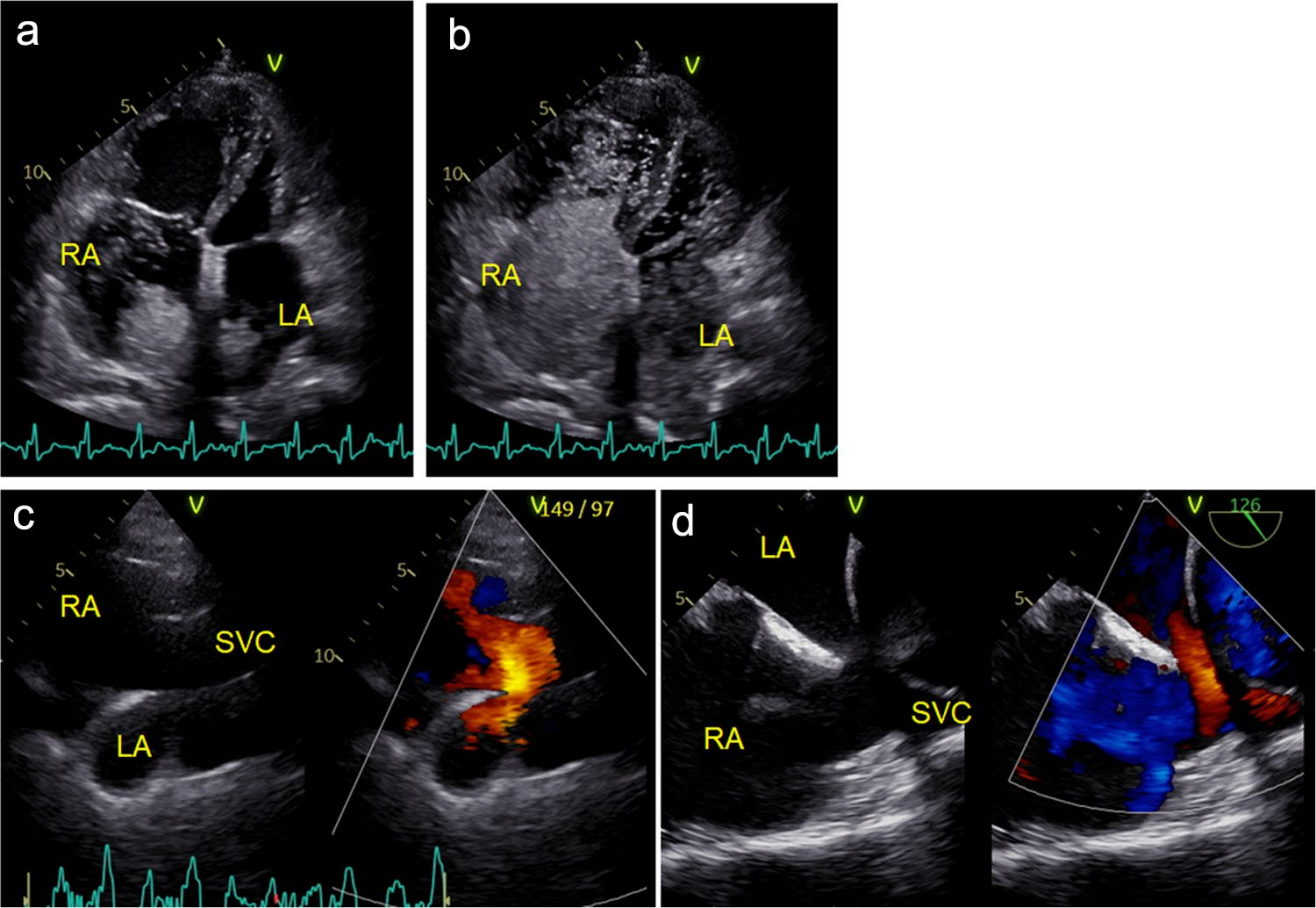
A case of superior sinus venosus-type ASD. **a**, **b** Apical four-chamber view of transthoracic echocardiography with agitated saline contrast. Agitated saline contrast injected from the right upper extremity appears and spreads simultaneously in the bilateral atria. **c** Visualization of the defect from the right sternal window. **d** Transesophageal echocardiographic image. *ASD* atrial septal defect, *LA* left atrium, *RA* right atrium, *SVC* superior vena cava

A typical condition presenting as RV dilatation is ASD. It is often diagnosed in adulthood after having been clinically asymptomatic. Once RV dilatation has been identified, the associated defect must be determined. In patients with secundum-type ASD, most of these defects can be diagnosed using TTE. The subcostal window is used because of the parallelism to the echo beam that can be provided by the shunt flow. However, the defect is generally visualized using the short-axis view of the aortic valve or the modified apical four-chamber view. The defect can be seen more closely with these views than with the subcostal window, and the diameter of the defect can be measured. Figure 2 shows the correlation between the maximum defect size on transesophageal echocardiography and the pulmonary to systemic blood flow ratio (Qp/Qs) in patients undergoing transesophageal echocardiography and right heart catheterization at our institution. Based on this graph, the maximum diameter is calculated to be 8.7 mm at a Qp/Qs of 1.5, 14.2 mm at a Qp/Qs of 2.0, 25.3 mm at a Qp/Qs of 3.0, and 36.4 mm at a Qp/Qs of 4.0. The data of patients with partial anomalous pulmonary venous return (orange dots) deviate from the approximate linear equation. In our experience, the defect diameters obtained via TTE are comparable to those obtained with transesophageal echocardiography, and therefore we should consider the probability of a coexisting anomality in cases with an atypical relationship between maximum diameter and Qp/Qs. We believe it is important to identify the ASD defect and consider the indications for intervention even if the patient has no symptoms. One reason is that a person who has undergone closure of the defect by the age of 24 can have the same outcomes as a healthy person [10]. Today, we can perform transcatheter defect closure in most patients with secundum-type ASD, so it is valuable to diagnose these patients. Although defects in patients with secundum-type ASD can be visualized on TTE, it is often difficult to detect in patients with other types of ASD. Sinus venosus-type ASD is often not diagnosed during routine echocardiographic examination, and it is helpful to perform it from the right sternal window or using agitated saline contrast. Figure 3 shows TTE images obtained using agitated saline contrast in a patient with superior sinus venosus-type ASD. Agitated saline contrast was injected via the right upper limb and appeared in both atria simultaneously, which is typical in patients with this type of ASD. In cases of right-sided chamber dilatation in which secundum-type ASD cannot be detected, TTE with agitated saline contrast should be performed after examination from the right sternal window.

### Post intracardiac repair for tetralogy of Fallot (TOF)

**Fig. 4:**
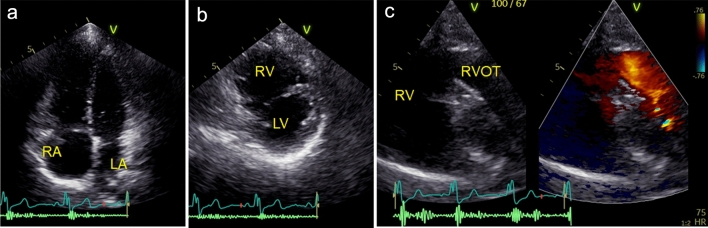
A case of severe pulmonary regurgitation following intracardiac repair for tetralogy of Fallot. **a** Apical four-chamber view. **b** Parasternal short-axis view. **c** Severe pulmonary regurgitation in RV outflow view. Dilatation of the RV relative to the LV is observed. Although the pulmonary valve is not visualized clearly enough to assess its characteristics, pulmonary regurgitation with wide jet convergence is observed. *LA* left atrium, *LV* left ventricle, *RA* right atrium, *RV* right ventricle, *RVOT* right ventricular outflow tract

The proportion of adult patients who have previously undergone surgery for TOF is high. They often have dilatation of the right-sided chambers due to significant pulmonary regurgitation or pulmonary stenosis. We should listen to the heart sounds with a stethoscope before the examination, because in most cases the pathological murmur of a pulmonic valve lesion can be clearly auscultated due to the proximity of the lesion to the chest wall. In some cases, we may not clearly visualize the pulmonary valve due to postoperative structural changes such as calcification and changes in the positions of various structures. However, pulmonic regurgitation jet flow into the RV is present in most cases. Therefore, vena contracta width, continuous wave Doppler, and flow reversal in pulmonic artery branches can be assessed [11]. The severity should be comprehensively evaluated on the basis of several parameters rather than just one. Similarly, when assessing pulmonary stenosis, the pulmonary valve jet velocity may underestimate the severity of stenosis for the reasons mentioned above. If the estimated RV systolic pressure from the tricuspid regurgitation (TR) pressure gradient is high and does not match the severity of the pulmonary stenosis, the pulmonary valve jet velocity may be underestimated. Based on the latest guidelines [12], the indication for intervention in asymptomatic patients with pulmonary regurgitation after intracardiac repair for TOF is considered based on progressive RV dilatation, RV systolic dysfunction, or an increase in RV systolic pressure greater than 80 mmHg. The values in the guidelines are based on CMR data, and the RV volumes calculated using 3D TTE are often smaller than those obtained with CMR [13]. However, estimation that takes into account its limitation may play an important role. Some patients may complain of symptoms even though their RV volumes are not large enough to be considered for intervention. The ratio of the RV to LV volume may be used to determine whether intervention is indicated [14]. Figure 4 shows TTE images of a symptomatic patient with severe pulmonary regurgitation after intracardiac repair for TOF. The RV end-diastolic volume calculated using 3D TTE was 134 mL, which is comparable to the value of 128 mL calculated using CMR, and the LV end-diastolic volume was 54 mL. Although the RV volume was not large enough to meet the guideline-recommended value of 160 mL/m^2^ [14], the ratio of the RV to LV volume was greater than 2. Therefore, this patient underwent re-RV outflow tract reconstruction. Based on previous published data [13] and our own experience, 3D echocardiography underestimates RV volume by about 10 mL compared to CMR.

### Tricuspid regurgitation

TR is a widely known cause of RV enlargement, and its incidence appears to be increasing due to the increased prevalence of long-standing persistent atrial fibrillation in the aging population [15]. Although it is easy to demonstrate the presence of TR, the need to rigorously assess its severity may be overlooked. In the latest Japanese Circulation Society guidelines [16], surgery for patients with severe TR associated with long-standing persistent atrial fibrillation is considered to be a class IIa indication if right-sided heart failure recurs, in which case transcatheter intervention will be introduced. The severity of TR should be assessed holistically based on multiple parameters, and severity beyond “severe” may be considered [17]. Detailed classification is needed to assess the results of transcatheter intervention for severe TR, and patients with TR considered worse than severe, i.e., massive TR, were reported to have a worse prognosis than those with severe TR [18]. In addition, patients with torrential TR, which is one grade worse than massive, were reported to have a worse prognosis than those with TR graded as massive or less [19]. When a patient with TR worse than severe is evaluated, RV contraction should be assessed simultaneously. Several clinical parameters, such as hemoglobin level, renal function, and RV end-systolic area determined using TTE, were reported to be predictors of cardiovascular events after surgery for severe TR [20, 21]. RV functional parameters are also important predictors of cardiovascular events. Tricuspid annular plane excursion (TAPSE) and RV free-wall strain were reported to be independent postoperative predictors of severe TR [22, 23]. Similar to the LV in patients with severe mitral regurgitation, the RV in patients with TR classified as severe or worse is in an unloaded condition, so it is important to keep in mind that RV contraction may be overestimated. There will often be situations in the near future in which it will be necessary to consider interventions for patients with TR that is severe or worse, and thus RV function should be assessed as well as RV severity.

## RV pressure overload

### Pulmonary hypertension

Pulmonary hypertension is a hemodynamic condition that is characterized by chronically elevated mean pulmonary artery pressure, and is classified into five categories [24]. The diagnosis of RV dilatation is a possibility in all categories except for group II. In the early stages of pulmonary hypertension, the RV is known to increase its contractility four- to five-fold in response to the increased vascular load. This can manifest as wall thickening and minimal RV dilatation, i.e., adaptive RV remodeling. As the disease progresses, the hypertrophic process may slow and stroke volume may decrease [25]. In addition, in a process known as maladaptive remodeling, the RV dilates to maintain stroke volume. RV dilatation has been reported to be associated with a worse prognosis, though increased wall thickness did not influence outcomes [26]. In the setting of RV dilatation in patients with pulmonary hypertension, RV contraction may already be impaired [27]. RV functional parameters predict the prognosis of patients with pulmonary hypertension, such as TAPSE and RV fractional area change. In the context of pulmonary hypertension, early identification of patients with a poor prognosis depends on the coupling of RV function and afterload [28]. TAPSE/pulmonary artery systolic pressure, as measured noninvasively using TTE, predicts both invasively measured RV-pulmonary artery coupling [29] and poor prognosis [29, 30].

## RV myocardial disease

### Arrhythmogenic RV cardiomyopathy

Arrhythmogenic right ventricular cardiomyopathy (ARVC) is an important disease that is associated with RV dilatation and is difficult to diagnose in its early stages. Its diagnosis is based on the task force criteria published in 2010 [31]. Since then, several authors reported the utility of adding other clinical factors to these criteria to formulate more appropriate follow-up strategies in relatives of probands with ARVC. These relatives are classified into three clinical stages: subclinical stage, defined by no signs or symptoms of disease; electrical stage, defined by electrical changes on electrocardiogram or Holter monitoring; and structural stage, defined by the presence of structural abnormalities [32]. Mast et al. analyzed RV echocardiographic parameters using 2D speckle tracking, and calculated the time of onset of shortening, post-systolic index, and systolic peak strain [33]. The authors assessed the severity of RV deformation abnormalities using these three parameters and classified relatives into three types: type I had normal deformation; type II had delayed onset of shortening and reduced peak systolic strain compared with type I; and type III had prominent systolic stretching and passive recoil or shortening during early diastole. The number of individuals with types I and II were comparable in the subclinical stage, while type II predominated in the electrical stage and type III was most common in the structural stage. Abnormal RV deformation patterns were found to already be present in the subclinical stage. Impaired contractility and increased stiffness were previously observed in the basal (subtricuspid) RV segment. In addition, disease progression during the 4-year follow-up period was extremely rare in the relatives with the subclinical stage who had no contractile abnormalities in the subtricuspid RV segment, and these individuals were considered to be in the true concealed stage [34]. Assessment focused on the basal RV segment may be helpful in the clinical setting when ARVC is suspected. The international criteria for arrhythmogenic cardiomyopathy (Padua criteria) developed in 2020 include three phenotypic variants: dominant right (classic ARVC), biventricular disease, and dominant left [35]. For each variant, echocardiographic findings, including those related to the LV, may be introduced.

### Cardiac sarcoidosis

Cardiac sarcoidosis sometimes results in RV lesions. Although there has been a recent tendency to assess the right-sided chambers in routine TTE examinations, it is difficult to verify RV involvement in cardiac sarcoidosis, which may often be differentiated from another cardiomyopathy such as ARVC. Phillips et al. reported that of 1140 patients with ARVC who were diagnosed based on guidelines published in 2010, 15 had cardiac sarcoidosis [36]. In their study, patients with cardiac sarcoidosis were significantly older and their incidence of symptomatic heart failure and atrioventricular block was higher than in those with typical ARVC. Regarding imaging findings, neither RV size nor function differed between the two groups, but LV function was slightly impaired in patients with cardiac sarcoidosis. Importantly, the incidence of late gadolinium enhancement on CMR, which reflects myocardial scarring of the septal wall, was higher in patients with cardiac sarcoidosis, though enhancement of the RV free wall was comparable. When RV involvement is assessed in cardiac sarcoidosis, ventricular septal findings should be evaluated simultaneously. RV involvement in this context was reported using TTE, especially 2D speckle tracking. Kusunose et al. reported a relationship between cardiac events and the results of biventricular 2D speckle tracking [37]. They found that basal LV longitudinal strain was useful in distinguishing patients with sarcoidosis and no cardiac involvement from normal controls. In addition, RV free wall strain and basal LV strain were analyzed as independent predictors of cardiovascular death and the development of cardiac involvement in sarcoidosis patients without cardiac involvement. Based on that report, latent biventricular myocardial injury may be present in sarcoidosis patients who have not been found to have cardiac lesions, and basal LV longitudinal strain is useful when sarcoidosis is suspected. It may be helpful to focus on the basal LV when RV involvement is suspected in cardiac sarcoidosis.

## Nonpathological RV dilatation

### Athlete’s heart

Dilatation of the RV is also a common finding in endurance athletes. Top-level athletes are often classified into endurance athletes and strength-trained athletes, and remodeling of the cardiac chambers differs between the two. The RV end-diastolic area was reported to be larger in both endurance and strength-trained athletes than in controls [38]. The diameters of the RV outflow and inflow tracts were largest in endurance athletes, but were comparable between strength-trained athletes and controls. Diagnosing athlete’s heart may be difficult due to similarities with ARVC. A study analyzing the absolute ranges of RV structural and functional parameters in endurance athletes reported that 28% of the athletes had RV outflow tract diameters greater than the major echocardiographic criteria for ARVC, and 83% met the minor criteria for a diagnosis of ARVC [39]. When indexed by body surface area, 6% of subjects still met the major criteria and 50% of them met the minor criteria. Nevertheless, estimation using the indexed value may be closer to the correct diagnosis. Despite recent concerns about the effects of intense exercise on the RV and other chambers, it has been reported that RV functional parameters are preserved in athletes. D’Andrea et al. [40] reported a cut-off point of -16% of RV global longitudinal strain to differentiate athlete’s heart from hypertrophic cardiomyopathy. Pagourelias et al. [38] reported that the RV fractional area change was higher in athletes than in normal controls, and that the value of RV global longitudinal strain was comparable between athletes and controls. Therefore, when differentiating between athlete’s heart and other RV cardiomyopathies, it is important to assess not only RV size but also RV function. RV dilatation in athletes is likely to be associated with concomitant LV remodeling, i.e., dilatation, and isolated RV enlargement should be suspected as a pathological process [41].

### Abnormalities of the thoracic cage

When TTE is performed in patients with pectus excavatum, it may be difficult to determine if the RV size is dilated and its function is preserved. A study using TTE and CMR to estimate ventricular chamber size and contraction in patients with pectus excavatum showed that 28% of patients had RV dilatation and 44% had impaired RV contraction [42]. In addition, pericardial effusion was observed in 56% of patients, possibly due to mechanical irritation of the pericardium. Although it may be difficult to differentiate these findings from those of athletes with RV dilatation and patients with ARVC, the characteristics of individuals with pectus excavatum should be kept in mind.

## Conclusions

RV dilatation is often initially detected using 2D TTE, and the differential diagnosis should be made by assessing RV function and the characteristics of related diseases.

## Abbreviations

ARVC: Arrhythmogenic right ventricular cardiomyopathy
ASD: Atrial septal defect
CMR: Cardiac magnetic resonance
LV: Left ventricle, left ventricular
Qp/Qs: Pulmonary to systemic blood flow ratio
RV: Right ventricle, right ventricular
TAPSE: Tricuspid annular plane systolic excursion
3D: Three-dimensional
TOF: Tetralogy of Fallot
TR: Tricuspid regurgitation
TTE: Transthoracic echocardiography, transthoracic echocardiographic
2D: Two-dimensional
